# Genetic Variants in *GAPDH* Confer Susceptibility to Sporadic Parkinson’s Disease in a Chinese Han Population

**DOI:** 10.1371/journal.pone.0135425

**Published:** 2015-08-10

**Authors:** Ling Liu, Nian Xiong, Ping Zhang, Chunnuan Chen, Jinsha Huang, Guoxin Zhang, Xiaoyun Xu, Yan Shen, Zhicheng Lin, Tao Wang

**Affiliations:** 1 Department of Neurology, Union Hospital, Tongji Medical College, Huazhong University of Science and Technology, Wuhan, Hubei, China; 2 Department of Neurology, the Second Affiliated Hospital of Wenzhou Medical University, Wenzhou, Zhejiang, China; 3 Department of Neurology, the Second Affiliated Hospital of Fujian Medical University, Quanzhou, Fujian, China; 4 Department of Psychiatry and Harvard NeuroDiscovery Center, Harvard Medical School and Laboratory of Psychiatric Neurogenomics, Division of Alcohol and Drug Abuse, McLean Hospital, Belmont, MA, United States of America; Second Affiliated Hospital, Zhejiang University, CHINA

## Abstract

**Background:**

Accumulating evidence has demonstrated that the glyceraldehyde-3-phosphate dehydrogenase (GAPDH) is a part of Lewy body inclusions and involves the pathogenesis of Parkinson’s disease (PD). However, it remains unknown whether or not genetic variation at the *GAPDH* locus contributes to the risk for PD.

**Methods:**

A total of 302 sporadic PD patients and 377 control subjects were recruited in our study for assessing two single nucleotide polymorphisms (rs3741918 and rs1060619) in the *GAPDH* gene. Both allelic association and additive models were used to analyze association between *GAPDH* variants and risk for PD.

**Results:**

Both polymorphisms were significantly associated with risk for PD after correction by Bonferroni multiple testing. The minor allele of rs3741918 was associated with decreased risk of sporadic PD (allelic contrast, OR = 0.74, 95% CI: 0.59–0.93, corrected *P* = 0.028; additive model, OR = 0.73, 95% CI: 0.58–0.92, corrected *P* = 0.018). While for the rs1060619 locus, the minor allele conferred increased risk for PD (allelic contrast, OR = 1.41, 95% CI: 1.14–1.75, corrected *P* = 0.007; additive model, OR = 1.43, 95% CI: 1.15–1.79, corrected *P* = 0.002).

**Conclusion:**

Our study indicates that *GAPDH* variants confer susceptibility to sporadic PD in a Chinese Han population, which is consistent with the role of GAPDH protein in neuronal apoptosis. To our knowledge, this is the first study of genetic association between *GAPDH* locus and risk for PD in the Chinese population.

## Introduction

Parkinson’s disease (PD) is a common progressive neurodegenerative disorder with a prevalence of ~1% in people over 60 years of age [1]. It is pathologically characterized by the loss of dopaminergic neurons in the substantia nigra together with the presence of proteinaceous cytoplasmic inclusions (Lewy bodies and Lewy neurites) [2]. Approximately 5–10% of the patients present an autosomal dominant or recessive mode of inheritance, while the majority of PD cases are apparently sporadic [3]. The etiology of sporadic PD is complex and multifactorial, involving aging, genetic and environmental risk factors. Over the years, candidate gene association studies have been extensively employed to identify loci where common variants contribute to the risk of PD. Most recently, a number of new susceptibility loci associated with PD have been identified through genome-wide association studies (GWAS) [4, 5].

The glyceraldehyde-3-phosphate dehydrogenase (*GAPDH*) gene, located at chromosome 12q13, encodes a widely expressed oxidoreductase. As a classical glycolytic enzyme, GAPDH displays a variety of novel biological functions besides its well-known role in glucose metabolism [6]. Intriguingly, recent studies indicate that GAPDH is involved in the pathogenesis of several neurodegenerative conditions such as Alzheimer’s disease (AD) and PD. S-nitrosylation of this protein under oxidative/nitrosative stress may induce neuronal apoptosis or dysfunction via nuclear translocation [7]. Specifically, GAPDH has been shown to interact with neurodegenerative disease-associated proteins, such as α-synuclein in PD, β-amyloid precursor protein in AD and huntingtin in Huntington’s disease [8–10]. Our previous study have indicated that rotenone, a common mitochondrial complex I inhibitor used to produce experimental parkinsonian models, could not only inhibit the glycolytic activity of GAPDH in dopamine neurons, but also lead to its overexpression and nuclear translocation as well as the formation of GAPDH-positive inclusions both *in vitro* and *in vivo* [11, 12].

In recent years, multiple genome-wide scans and linkage analyses have identified *GAPDH* locus for susceptibility to late-onset AD (LOAD) [13]. Subsequently a series of association studies have demonstrated the association of *GAPDH* with risk for LOAD [13–16]. However, the correlation between the *GAPDH* gene and PD remains unclear. As we all know, neurodegenerative disorders like AD and PD share many similarities in the etiology and pathogenesis pathways [17]. Recently several reports have implied some possibility of a pathological overlap between AD and PD [18, 19]. Given such strong evidence showing that GAPDH protein contributes to the pathogenesis of PD and AD, along with the association of *GAPDH* with LOAD, we reasonably speculate that *GAPDH* variants may be related to PD. Thus, here, we conducted a case-control study to investigate the association of *GAPDH* variants with susceptibility to sporadic PD in a Chinese Han Population.

## Materials and Methods

### Study population

This study, consisting of 302 sporadic PD patients and 377 healthy control subjects, was approved by the Ethics Committee of Tongji Medical College, Huazhong University of Science and Technology (HUST). Written informed consent was obtained from each of these participants or their legal guardians. Patients from Hubei province were recruited from the Department of Neurology, Union Hospital, HUST. The PD diagnosis was made according to the UK PD Society Brain Bank clinical diagnostic criteria by two or more experienced neurologists. Sporadic PD was defined as PD without a family history of disease. The mean age at onset of PD was 58.0 ± 12.8 years, ranging from 19 to 87 years. A total of 377 healthy volunteers matched for sex, age, and area of residence were recruited as controls (mean age 56.8 ± 12.5 years, ranging from 17 to 90 years). All cases and controls were unrelated Han Chinese.

### Polymorphism genotyping

Two single nucleotide polymorphisms (SNPs), rs3741918 and rs1060619 within intron 2 of *GAPDH* gene, were selected for genotyping (rs3741918 at position 6535090 and rs1060619 at position 6535615 in chromosome 12). These two SNPs, including rs1060619 as one of tagging SNPs in *GAPDH*, have previously been identified and reported for significant association with LOAD, with minor allele frequencies greater than 0.2 in the HapMap Asian populations. We genotyped them by means of direct sequencing. Genomic DNA was extracted from the peripheral blood of the subjects using the Blood Genomic DNA Kit (BioTeke Corporation, Beijing, China) according to the manufacturer’s instructions. Polymerase Chain Reaction (PCR) was performed using forward primer 5’-GGGTCTTTGCAGTCGTATGGG-3’ and reverse primer 5’-AATCAGGAGTGGGAGCACAGGT-3’ to obtain product for Sanger sequencing. The PCR amplification system included 60 ng of genomic DNA, 15 μl 2 × DreamTaq Green PCR Master Mix (containing DreamTaq DNA Polymerase, dNTPs, MgCl_2_ and PCR buffer; Thermo Fisher Scientific Inc, Waltham, Massachusetts, USA) and 2 μl of primer mix, to give a total reaction volume of 30 μl. The amplification reaction consisted of an initial step at 95°C for 5 minutes, 35 cycles of denaturation at 95°C for 30 seconds, annealing at 62°C for 30 seconds, and extension at 72°C for 1 minute, with a final step at 72°C for 10 minutes. PCR amplification was performed using GeneAmp PCR 9600 thermocycler (Applied Biosystems Inc, Foster, California, USA). Then the PCR products were loaded on the ABI 3730xl DNA Analyzer (Applied Biosystems Inc, Foster, California, USA) for the genotyping. Approximately 5% random samples were re-sequenced to confirm the genotyping results. The DNA sequences were analyzed using Chromas, Version 2.2.3 (Technelysium Pty Ltd, Brisbane, Queensland, Australia). The average genotype call rate for the tested SNPs was >99%.

### Statistical analysis

Sex and age frequencies between patients and controls were compared using a Pearson’s χ^2^ test and an independent-sample t-test, respectively. Assessment for deviation from Hardy-Weinberg equilibrium was performed using a goodness-of-fit χ^2^ test in case and control group. Allelic association and genotypic association assuming additive genetic model between cases and controls for each SNP were analyzed using a Pearson’s χ^2^ test. The final *P* values were adjusted by Bonferroni correction for multiple testing considering two loci and two association models. A value of *P*<0.05 (two-tailed) was considered statistically significant. Odds ratio (OR) and 95% confidence interval (95% CI) were calculated to estimate the risk of PD associated with each SNP. All the statistical analyses above were conducted by SPSS v20.0 statistical packages (IBM Corporation, New York, USA). Power calculations were performed using the Power and Sample Size Calculation (PS) program (http://biostat.mc.vanderbilt.edu/wiki/Main/PowerSampleSize).

## Results

The demographic characteristics of 302 PD patients and 377 control subjects are shown in Table 1. The cases and controls were well matched on the distribution of sex and age (*P* >0.05). The genotype frequencies of the tested SNPs in both cases and controls showed no significant departure from the Hardy-Weinberg equilibrium. Power analysis showed that the study could provide power between 0.468–0.602 for the tested SNPs under an allelic genetic model.

**Table 1:** Basic characteristics of study subjects.

Features	Cases (n = 302)	Controls (n = 377)	*P*-value
	N (%)	N (%)	
sex			
Male	186 (61.6)	230 (61.0)	0.877
Female	116 (38.4)	147 (39.0)	
Age (years)	58.0 ± 12.8	56.8 ± 12.5	0.209
≤50, N (%)	42.6 ± 6.1, 94 (31.1%)	42.4 ± 6.0, 132 (35.0%)	0.809
>50, N (%)	65.0 ± 8.1, 208 (68.9%)	64.6 ± 6.9, 245 (65.0%)	0.532

The distributions of genotypic and allelic frequencies of the two SNPs in cases and controls are presented in Table 2. Both SNPs were significantly associated with sporadic PD after correction by Bonferroni multiple testing. When using an allelic association model, we found that the minor allele A of rs3741918 tended to reduce the risk of developing PD (OR = 0.74, 95% CI: 0.59–0.93, corrected *P* = 0.028). While for the rs1060619 locus, the minor allele T increased risk of PD by 1.41-fold compared with the C allele (95% CI: 1.14–1.75, corrected *P* = 0.007). Similarly, significant genotypic associations were observed under an additive model. The minor allele of rs3741918 was associated with decreased risk of PD (A vs. T, OR = 0.73, 95% CI: 0.58–0.92, corrected *P* = 0.018), while the minor allele of rs1060619 conferred increased risk for sporadic PD (T vs. C, OR = 1.43, 95% CI: 1.15–1.79, corrected *P* = 0.002).

**Table 2:** Genotypic and allelic associations of *GAPDH* variants with PD.

SNP	Cases (n = 302)	Controls (n = 377)	OR (95% CI)	*P*-value	*P*-value^a^
	N (%)	N (%)			
rs3741918					
Genotype					
AA	34 (11.3)	56 (14.9)			
AT	139 (46.0)	199 (52.8)			
TT	129 (42.7)	122 (32.4)			
Additive model			0.73 (0.58–0.92)	0.005	0.018
Allele					
A	207 (34.3)	311 (41.2)	0.74 (0.59–0.93)	0.007	0.028
T	397 (65.7)	443 (58.8)			
rs1060619					
Genotype					
TT	95 (31.5)	78 (20.7)			
CT	148 (49.0)	201 (53.3)			
CC	59 (19.5)	98 (26.0)			
Additive model			1.43 (1.15–1.79)	4.17×10^−4^	0.002
Allele					
T	338 (56.0)	357 (47.3)	1.41 (1.14–1.75)	0.002	0.007
C	266 (44.0)	397 (52.7)			

^a^ Adjusted by Bonferroni correction for multiple testing.

## Discussion

We conducted this case-control study in a Chinese Han population as isolated populations are generally more powerful for disease association mapping than mixed groups [20]. Our data indicated that both rs3741918 and rs1060619 within *GAPDH* were associated with PD. The minor allele of rs3741918 was a relatively protective allele, while the minor allele of rs1060619 conferred higher risk for PD. To our knowledge, this is the first study to explore genetic association between *GAPDH* locus and risk for PD in the Chinese population. In addition, we have performed HapMap database query in Asian populations including Han Chinese in Beijing and Japanese in Tokyo, and acquired linkage information between rs1060619 and SNPs within the gene, SNPs upstream and downstream of the gene, and SNPs in adjacent genes. We found strong linkage disequilibrium (LD) between rs1060619 and rs6489721 within the *GAPDH* promoter region (r^2^ = 0.82), or rs1060619 and rs2886093 in intron 2 of *GAPDH* (r^2^ = 0.87), thus representing a larger region of association. Interestingly, our group has also reported significant association between PD and *NCAPD2* which is closely adjacent to *GAPDH* locus [21].

Not only is GAPDH a critical glycolytic enzyme in cellular energy metabolism, but also involved in many other cellular processes including neurodegeneration. Recent studies have indicated that GAPDH protein may play an important role in dopaminergic neuronal apoptosis. Studies on both cell models and post mortem brain tissues from sporadic PD have confirmed that GAPDH co-aggregates with α-synuclein which is the primary component of Lewy inclusions [10, 22]. In several *in vitro* apoptotic models induced by various neurotoxins including MPP +, 6-OHDA and rotenone, abnormal aggregation and nuclear translocation of this protein were consistently observed [11, 23–25]. Furthermore, GAPDH knockdown reduced the cytotoxicity of these neurotoxins on dopaminergic cells [11, 23]. Importantly, a recent study showed that substitution of cysteine for serine-284 of human GAPDH led to aggregate-prone GAPDH, and that its expression in SH-SY5Y cells resulted in greater oxidative stress-linked cell death than expression of wild type-GAPDH [26]. These findings suggest consistently that alteration in GAPDH function derived from genetic variation might be implicated in the pathogenesis of PD.

Recently a few association studies have indicated that *GAPDH* and its paralogs including *p-GAPD* and *GAPDHS* were implicated in LOAD, whereas the strength and direction of association have not been consistent [15]. In the original report, Li *et al* found that two variants in *GAPDH*, rs3741916 and rs1060621, conferred susceptibility to LOAD in two independent samples and combined series [14]. Subsequently, Lin *et al* identified a haplotype block (rs1060621- rs1060620- rs1060619) within this gene significantly associated with LOAD [16]. In another follow-up study, Lee *et al* found that haplotype C-A-T at SNPs rs3741916- rs3741918- rs1060620 was associated with LOAD in a case-control and a family datasets [17]. These results supported the presence of LOAD variants and heterogeneity at the *GAPDH* locus.

Recent meta-analysis of GWAS generated for populations of European descent from the PDGene database indicated that there was no significant association between *GAPDH* and PD. However, allele frequencies of the genotyped SNPs in European ancestry populations are distinct from those in Asian populations. The details on the minor allele frequencies of the two SNPs in populations from Europe, Asia (including Han Chinese in Beijing and Japanese in Tokyo) and our cohort are as follows: rs1060619, 0.233 (C), 0.492 (T), 0.473 (T); rs3741918, 0.225 (A), 0.4 (A), 0.412 (A), respectively. As we can see, allele frequencies of the two variants in Chinese population, which are very similar to those in Japanese though, significantly differ from those in Europeans. To our knowledge, until now, there is no report on the association of *GAPDH* with PD in Asian populations. Our data, for the first time, provide positive evidence to support a novel finding that *GAPDH* variants are associated with sporadic PD in a Chinese Han population, which is consistent with the role of GAPDH protein in neuronal apoptosis. However, it is noteworthy that there is a lack of association in the GWAS of Japanese patients, suggesting that either the association is specific to Han Chinese or a false positive finding in which case the observed associated SNPs might be in LD with a disease-predisposing variant. Our observation merits further investigation, especially replication in additional, larger and independent cohorts, both in Chinese and other ethnic populations. Functional study at the cellular level is also required to ascertain whether they are true disease-predisposing variants.

Some limitations in this study must be noted. A common caveat in case-control study is population stratification, which may be a confounding factor for association analysis and cause false positive results. For the study design, we limited all subjects’ ethnicity to Han Chinese and collected controls from the same geographical area as cases in central China. Also, our patients and control subjects were well matched for sex and age. Thus, there was no substantial population admixture in our cohort, although this could not be completely excluded. Another limitation of the study is the limited sample size (302 patients and 377 control subjects), which did not allow stratified analysis by age or sex. In that case, subgroup analysis may not be able to provide adequate power to detect the effect of these SNPs.

In summary, our study provides positive evidence to support a novel finding that *GAPDH* is associated with sporadic PD in a Chinese population. Further studies are required to validate the finding in more populations and to elucidate the mechanistic role of *GAPDH* variants in PD susceptibility.
